# Antithrombin controls tumor migration, invasion and angiogenesis by inhibition of enteropeptidase

**DOI:** 10.1038/srep27544

**Published:** 2016-06-08

**Authors:** Ginés Luengo-Gil, María Inmaculada Calvo, Ester Martín-Villar, Sonia Águila, Nataliya Bohdan, Ana I. Antón, Salvador Espín, Francisco Ayala de la Peña, Vicente Vicente, Javier Corral, Miguel Quintanilla, Irene Martínez-Martínez

**Affiliations:** 1 Servicio de Hematología y Oncología Médica, Hospital Universitario Morales Meseguer, Centro Regional de Hemodonación, Universidad de Murcia, IMIB-Arrixaca, Murcia, Spain; 2 Instituto de Investigaciones Biomédicas Alberto Sols, CSIC-UAM, Madrid, Spain

## Abstract

Antithrombin is a key inhibitor of the coagulation cascade, but it may also function as an anti-inflammatory, anti-angiogenic, anti-viral and anti-apoptotic protein. Here, we report a novel function of antithrombin as a modulator of tumor cell migration and invasion. Antithrombin inhibited enteropeptidase on the membrane surface of HT-29, A549 and U-87 MG cells. The inhibitory process required the activation of antithrombin by heparin, and the reactive center loop and the heparin binding domain were essential. Surprisingly, antithrombin non-covalently inhibited enteropeptidase, revealing a novel mechanism of inhibition for this serpin. Moreover, as a consequence of this inhibition, antithrombin was cleaved, resulting in a molecule with anti-angiogenic properties that reduced vessel-like formation of endothelial cells. The addition of antithrombin and heparin to U-87 MG and A549 cells reduced motility in wound healing assays, inhibited the invasion in transwell assays and the degradation of a gelatin matrix mediated by invadopodia. These processes were controlled by enteropeptidase, as demonstrated by RNA interference experiments. Carcinoma cell xenografts in nude mice showed *in vivo* co-localization of enteropeptidase and antithrombin. Finally, treatment with heparin reduced experimental metastasis induced by HT29 cells *in vivo*. In conclusion, the inhibition of enteropeptidase by antithrombin may have a double anti-tumor effect through inhibiting a protease involved in metastasis and generating an anti-angiogenic molecule.

Antithrombin is the main inhibitor of the coagulation cascade, but it has many other roles outside of hemostasis. Different studies have determined that antithrombin has an anti-inflammatory role by inhibiting nuclear factor KB signaling^1^,^2^. Moreover, results from clinical trials have supported the anti-inflammatory properties of antithrombin in patients with sepsis or disseminated intravascular coagulation^3^,^4^. Angiogenesis is another function identified for antithrombin^5^. However, only the latent and cleaved non-inhibitory conformations of antithrombin have anti-angiogenic properties. Thus, latent antithrombin reduces the vascularization of fibrosarcoma^6^. The anti-angiogenic effects of cleaved and latent antithrombin have been compared to those of inhibitors such as endostatin and TNP-470^7^. Antithrombin may also function as an anti-viral molecule by altering the expression of several genes through different signaling pathways related to hepatitis C virus infection^8^. Moreover, antithrombin is involved in other cellular processes, such as apoptosis^9^, in which it exerts a protective role in mouse models of liver injury through inhibition of an unknown protease^10^. On the basis of all reported functions, antithrombin can be defined as a pleiotropic protein. To date, antithrombin has not been associated with cancer, although proteolytic cascades are involved in tumorigenesis. Tumors grow and metastasize by means of various proteolytic activities in conjunction with intra- and inter-cellular signaling events. For example, to invade surrounding tissues and spread to distant locations, tumor cells exploit the activity of deregulated proteases to degrade and remodel intercellular junctions and extracellular matrix interactions. Members of the superfamily of serine proteases known as type II transmembrane serine proteases (TTSPs) are among the enzymes deregulated during tumor growth and progression. These TTSPs include TMPRSS2, corin, hepsin, enteropeptidase or TMPRSS15, matriptase, TMPRSS3, TMPRSS4, matriptase-2 and differentially expressed in squamous cell carcinoma gene 1 (DESC1)^11^. Recently, it has been shown that protein C inhibitor (PCI) and, to a lesser extent, antithrombin, may regulate the function of enteropeptidase. However, PCI does not inhibit enteropeptidase *in vivo*^12^. In this study, we characterized the antithrombin-mediated inhibition of enteropeptidase and investigated the effects of antithrombin on tumor cell migration, invasion and angiogenesis. Besides, we assessed the effect of heparin treatment in metastasis in an *in vivo* mouse model.

## Results

### Antithrombin-mediated inhibition of enteropeptidase in cancer cells

We first analyzed enteropeptidase gene (*TMPRSS15*) expression by qRT-PCR in the following cancer cell lines: MDA-MB231 (breast), MCF7 (breast), A549 (lung), U-87 MG (glioblastoma-astrocytoma), KATO-III (stomach), HT-29 (colon), and CACO-2 (colon). Only the HT-29, A549 and U-87 MG cell lines expressed *TMPRSS15*; therefore, they were selected for subsequent studies (Supplementary Fig. S1). None of these cell lines expressed antithrombin.

**Figure 1:**
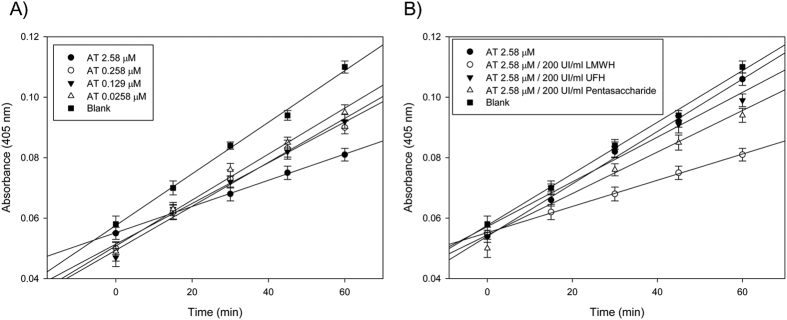
Enteropeptidase activity measured after solubilization from U-87 MG cells. (**A**) Residual activity of enteropeptidase after incubation of cells with different concentrations of antithrombin activated by 200 U/mL low molecular weight heparin. (**B**) Effect of different heparins (UFH: unfractionated heparin; and LMWH: low molecular weight heparin) on the inhibition of enteropeptidase activity by antithrombin. The values are represented as the mean of three different experiments.

Next, we analyzed the effect of antithrombin on the activity of enteropeptidase in U-87 MG cells. We observed that antithrombin inhibited enteropeptidase activity in a concentration-dependent manner (Fig. 1A). This inhibition was stimulated by the binding of heparin (Fig. 1B). Moreover, low molecular weight heparins (LMWH) stimulated antithrombin more efficiently than did unfractionated heparins or pentasaccharide (Fig. 1B). Figure 1 shows the results obtained with U-87 MG cells, and experiments performed with HT-29 and A549 cells rendered similar results. Heparins had no effect on the activity of enteropeptidase (Fig. 1A, blank).

Antithrombin requires the following two functional domains to inhibit its target proteases in the coagulation cascade: the residue R393 or P1 at the reactive center loop^13^,^14^ and the heparin binding domain^15^,^16^. To identify functional domains of antithrombin involved in enteropeptidase inhibition, two variants were tested. Antithrombin London lacks R393 at the reactive center loop, whereas antithrombin Toyama (R47C) has reduced heparin affinity. As shown in Table 1, both variants exhibited significantly impaired or abolished anti-enteropeptidase activity. Antithrombin London was completely unable to inhibit enteropeptidase. However, antithrombin Toyama showed a reduced inhibitory effect because its reactive center loop is intact, but its activation by heparin is deficient. Table 1 shows the results obtained with U-87 MG cells. Experiments performed with HT-29 and A549 cells yielded similar results.

### Antithrombin inhibition of purified enteropeptidase

**Figure 2:**
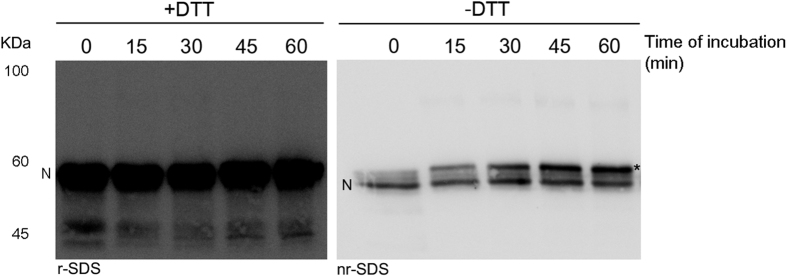
Electrophoretic evaluation of the interaction between antithrombin and enteropeptidase at different incubation times. SDS-PAGE was run under reducing (+DTT, r-SDS) and non-reducing conditions (−DTT, nr-SDS), and western blotting with immunodetection with antithrombin-specific antibody was carried out. An asterisk represents cleaved antithrombin. N indicates the electrophoretic mobility in SDS for antithrombin in its native conformation.

The interaction between the protease and inhibitor was verified by SDS-PAGE. Intriguingly, although time course experiments were performed to follow the formation of covalent complexes, no bands were detected at the expected molecular mass (90 kDa approximately) (Fig. 2, left panel). However, enteropeptidase activity was completely inhibited, because no hydrolysis of its chromogenic substrate was detected after incubation of the cells with antithrombin primed with LMWH. The resolution of these samples under non-reducing conditions provided an explanation: increasing amounts of cleaved antithrombin were detected in this time course incubation (Fig. 2, right panel). Cleaved antithrombin usually migrates with lower mobility than intact protein^17^,^18^. Moreover, N-terminal sequencing of the band detected under non-reducing conditions after 1 hour of incubation with enteropeptidase confirmed that cleavage occurred at R393, the same residue recognized by the target enzymes of antithrombin involved in coagulation^14^. The cleavage at R393 may be detected as the C-terminal peptide of antithrombin remains bound after cleavage due to the disulfide bond between Cys247 and Cys430^17^. The cleavage of antithrombin was also verified in the supernatant of cells after 1 hour of incubation with antithrombin, as described in Materials and Methods (Supplementary Fig. S2). The formation of covalent complexes between enteropeptidase and antithrombin was not detected in these assays.

### Enteropeptidase-mediated cleavage of antithrombin inhibits vessel formation by endothelial cells

**Figure 3:**
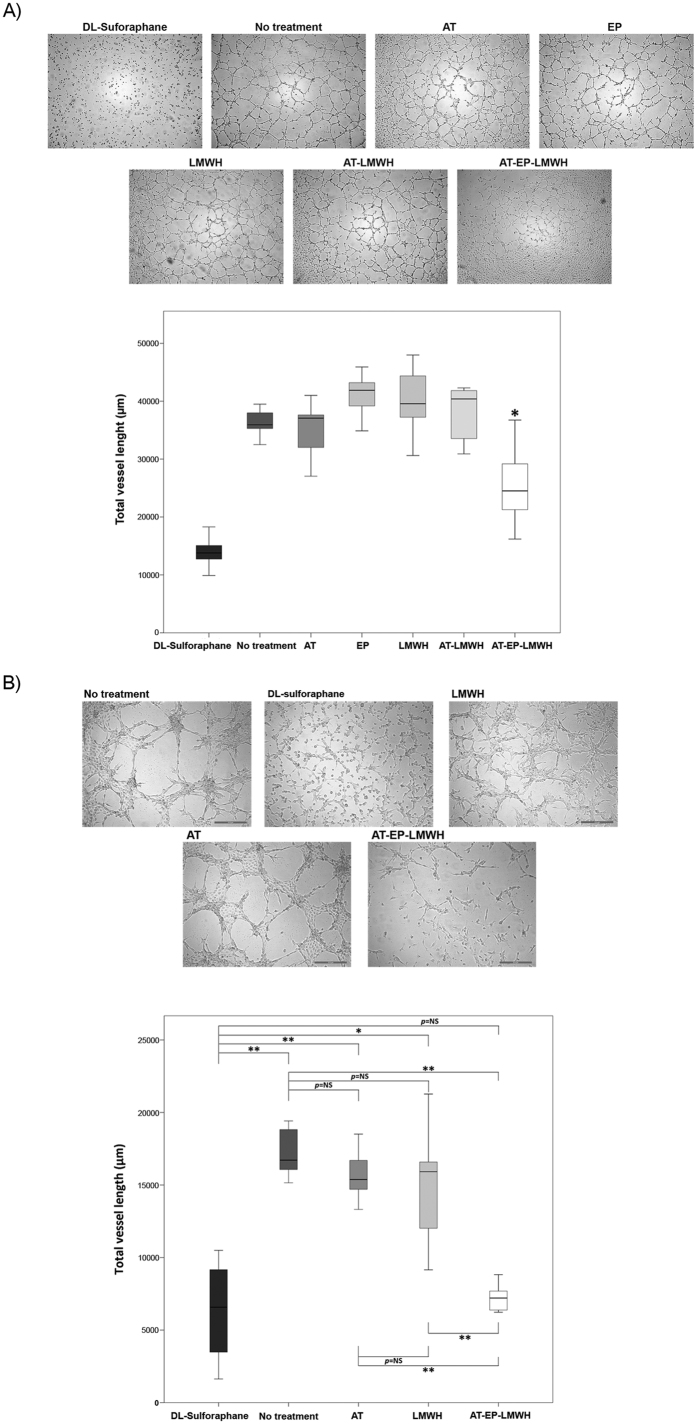
Vessel formation by endothelial EA.hy926 cells and co-culture of EA. hy926 and U-87 MG cells on a Matrigel layer. Vessel formation was evaluated in different conditions after 24 hours of incubation. (**A**) Vessel formation by endothelial cells. (**B**) Vessel formation by co-culture of endothelial and U-87 MG cells. D,L-sulforaphane was used as positive control. Images were recorded with a Leica microscope at 5×, and ImageJ was used to analyze angiogenesis. Statistical analysis was carried out with a Mann-Whitney U test. Each condition was evaluated in octuplicate. *p < 0.05; **p < 0.01. AT: antithrombin, LMWH: low molecular weight heparin; AT-EP-LMWH: mix of antithrombin, enteropeptidase and low molecular weight heparin after 24 hours of incubation, when enteropeptidase is completely inhibited and antithrombin is completely cleaved.

Because cleaved antithrombin has anti-angiogenic properties^5^, we investigated whether enteropeptidase-cleaved antithrombin was able to inhibit the formation of capillary-like structures by endothelial cells seeded in Matrigel-coated plates. The formation of enteropeptidase-cleaved antithrombin was first confirmed electrophoretically (Supplementary Fig. S3). Cleaved antithrombin (AT-EP-LMWH) inhibited vessel formation by EA.hy926 cells in Matrigel, whereas antithrombin, enteropeptidase or LMWH alone had no effect (Fig. 3A). This effect was also evaluated in a co-culture of EA.hy926 and U-87 MG cells. Although the number of vessels was reduced in comparison with those established by the endothelial cells on their own, addition of cleaved antithrombin (AT-LMWH-EP) resulted in the most significant reduction of vessel formation in the co-culture, confirming its anti-angiogenic effect (Fig. 3B, Supplemental Table S2). D,L-sulforaphane was used in this assay as a positive control. We also confirmed that EA.hy926 cells do not express enteropeptidase (Supplementary Fig. S4).

### Antithrombin inhibits cancer cell migration and matrix degradation through enteropeptidase in cancer cells

Proliferation, migration and invasion are deregulated in cancer cells. We sought to investigate the potential role of the antithrombin-mediated inhibition of enteropeptidase in these three processes. Antithrombin had no effect on HT-29, A549 and U-87 MG cell proliferation regardless of whether it was activated by LMWH (Supplementary Fig. S5).

**Figure 4:**
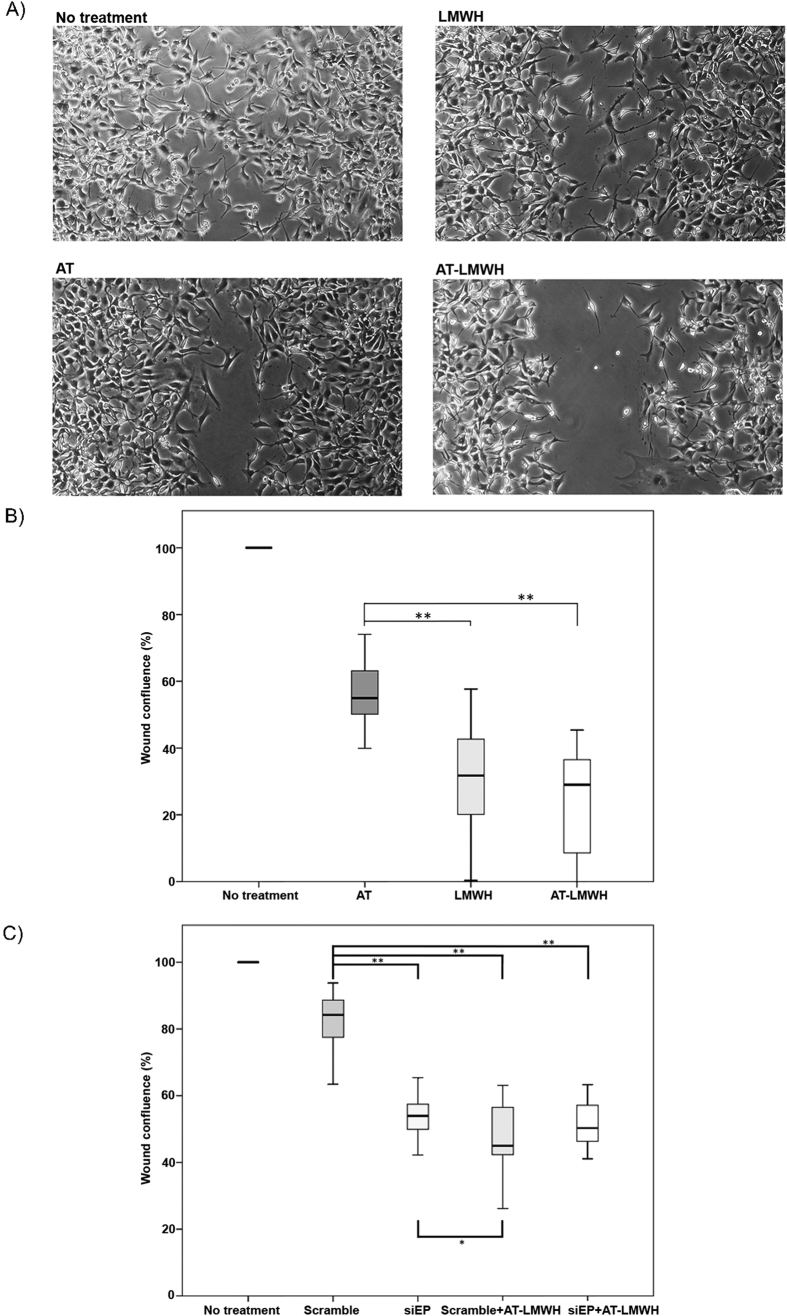
Effects of antithrombin and heparin on cell migration of U-87 MG cells. Wound healing was evaluated after incubation of cells for 24 hours without treatment and after incubation with low molecular weight heparin (LMWH), antithrombin (AT) or antithrombin and LMWH in combination (AT-LMWH). (**A**) Microscope images of cells 24 hours after the wound with the pipette tip. (**B**) Percentage of wound confluence under the different conditions. (**C**) Percentage of wound confluence under the different conditions in *TMPRSS15*-silenced cells. Each condition was evaluated in triplicate, and five different images were processed for each different assay; *p < 0.05; **p < 0.01. Images were recorded with a Leica microscope at 5×, and Image J was used to analyze migration. Statistical analysis was carried out with a Mann-Whitney U test.

Next, we investigated the effect of antithrombin on cell migration using an *in vitro* wound healing assay^19^. A549 and U-87 MG cells were selected for these experiments because HT-29 had a reduced migration, even in the absence of treatment (Supplementary Fig. S6A). As shown in Fig. 4A,B and Supplementary Fig. S6B,C, A549 and U-87 MG cells had an impaired ability to close the wound in the presence of either antithrombin or LMWH alone, but the effect on cell migration was more pronounced after combined treatment with antithrombin and LMWH. Downregulation of enteropeptidase expression by siRNA treatment reduced U-87 MG cell motility to the same extent as did antithrombin (Fig. 4C). These data are consistent with the involvement of enteropeptidase in tumor cell migration and with a heparin-enhanced inhibitory effect of antithrombin on cell migration, likely mediated by modulation of enteropeptidase activity.

**Figure 5:**
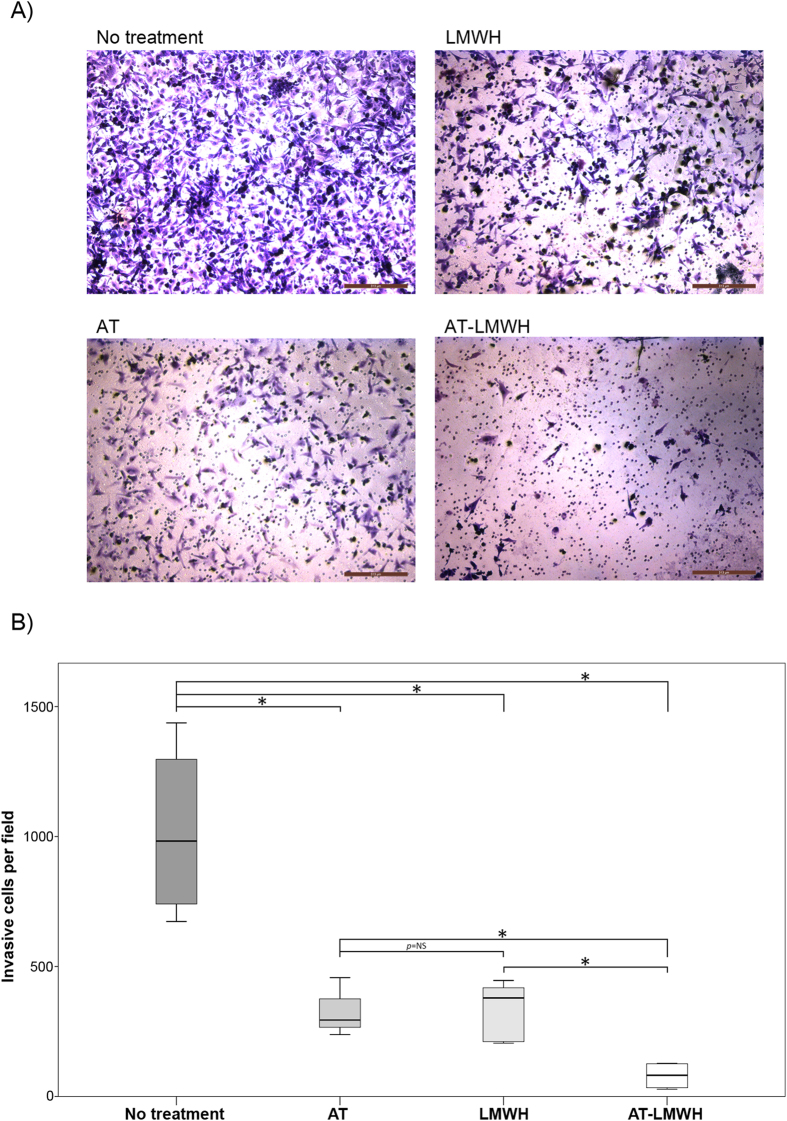
Effects of antithrombin and heparin on invasion by a transwell assay. Cell invasion was evaluated after incubation of U-87 MG cells for 6 hours under the following conditions: no treatment, incubation with low molecular weight heparin (LMWH), antithrombin (AT) or incubation with antithrombin and LMWH in combination (AT-LMWH). (**A**) Microscope images of cells invaded after 6 hours of incubation. (**B**) Percentage of cells invading under the different conditions. Each condition was evaluated in triplicate, and three different images were processed for each different assay; *p < 0.05; **p < 0.01. Images were recorded with a Leica microscope at 5×, and Image J was used to analyze cells that had migrated. Statistical analysis was carried out with a Mann-Whitney U test.

**Figure 6:**
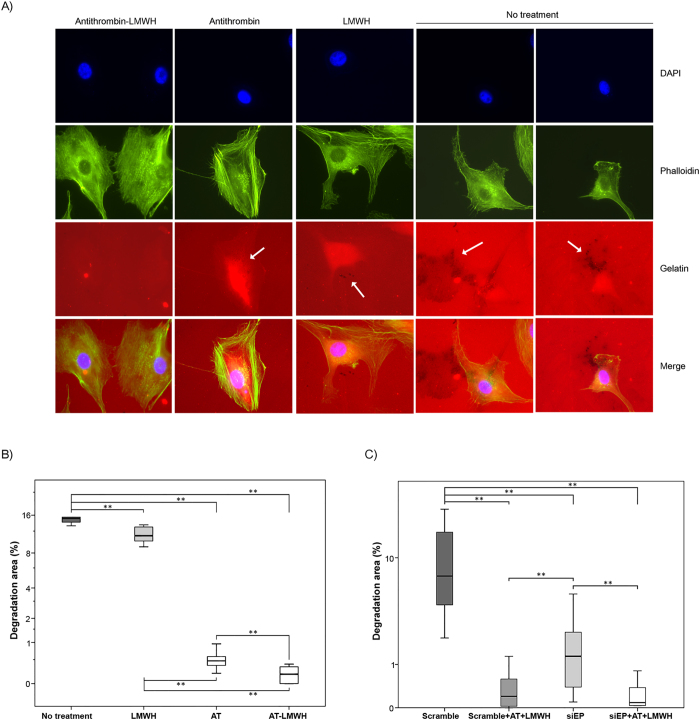
Effects of antithrombin and heparin on invadopodia degradation of gelatin coated coverslips. (**A**) Representative micrographs of U-87 MG cells on a rhodamine-gelatin matrix. Cells were incubated without treatment or with low molecular weight heparin (LMWH), antithrombin (AT) and antithrombin activated by LMWH (AT-LMWH). Invadopodia were identified by colocalization of actin punctae over or close to areas of degraded matrix, which are indicated with a white arrow. (**B**) Quantification of invadopodia-mediated gelatin degradation of cells under the different treatments. (**C**) Quantification of invadopodia-mediated gelatin degradation of cells transfected with a scrambled siRNA or the specific silencer for *TMPRSS15* gen (SiEP) with or without treatment with AT in combination with LMWH. Each condition was assayed in triplicate, and up to twenty-five different images were processed for each condition; *p < 0.05; **p < 0.01. Images were acquired with a Nikon 90i microscope at 60×, analyzed with Fiji software to calculate gelatin degradation and statistical analysis was carried out with a Mann-Whitney U test.

We also evaluated the role of enteropeptidase on invasion and the effect of antithrombin during this process. A Matrigel invasion assay was performed with U-87 MG and A549 cells (Fig. 5 and Supplementary Fig. S7). As shown in the Figures, a significant reduction of invasiveness was observed when cells were treated with antithrombin activated by LMWH. Additionally, it has previously been demonstrated that invadopodia, ventral actin-rich plasma membrane protrusions involved in tumor cell invasion, contain metalloproteases and serine proteases that are responsible for matrix degradation^20^. Because U-87 MG cells are able to form invadopodia, we also analyzed the effect of antithrombin on the formation and activity of these structures. Interestingly, treatment with antithrombin significantly impaired the ability of the cells to degrade gelatin without affecting the formation of actin puncta on the basal surface of U-87 MG cells, an effect that was enhanced by LMWH (Fig. 6A,B). Knockdown of enteropeptidase significantly reduced gelatin degradation (Fig. 6C). Interestingly, treatment of enteropeptidase-downregulated cells with antithrombin plus LMWH further reduced gelatin degradation activity. This result might suggest that other antithrombin target proteases are involved in addition to enteropeptidase, because invadopodia contain a machinery of proteases responsible for complete extracellular matrix degradation^20^.

### Antithrombin and enteropeptidase co-localize in tumors induced *in vivo*

**Figure 7:**
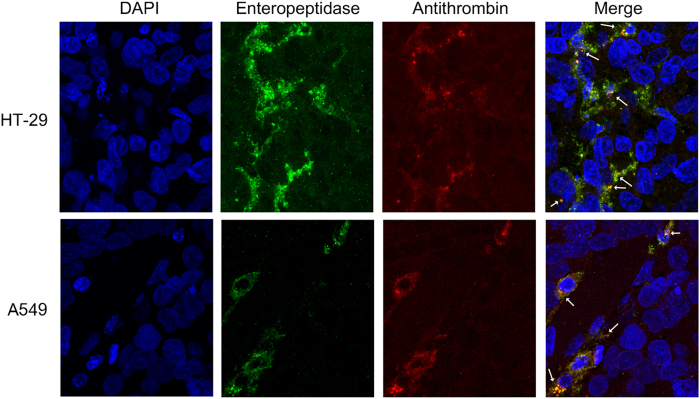
Co-localization of enteropeptidase and antithrombin in human xenograft tumors surgically removed from nude mice. HT-29 and A549 tumor cells were subcutaneously injected in the two flanks of nude mice and removed when they reached approximately 1 cm in diameter. Immunohistofluorescence of slides from tumors was performed by incubating with Alexa Fluor 549 and Alexa Fluor 488 antibodies for detecting the primary antibodies specific to enteropeptidase and antithrombin, respectively. Some of the areas of co-localization are indicated with white arrows.

To ascertain whether antithrombin and enteropeptidase interact *in vivo*, we analyzed their co-localization in tumors induced by injection of HT-29 and A549 cell lines into nude mice. Only these cell lines were used because U-87 MG cells were not tumorigenic (Supplementary Table S1). As shown in Fig. 7, mouse antithrombin co-localized with enteropeptidase in tumor cells, indicating a potential interaction between both molecules; antithrombin may extravasate to the tumor tissue (Supplementary Fig. S8). Although co-localization of both proteins is not sufficient to support the anti-tumor role of antithrombin, the colocalization of both proteins *in vivo* strongly suggests that antithrombin-mediated inhibition of enteropeptidase may be physiologically relevant.

### Heparin treatment reduces HT29-induced experimental metastasis *in vivo*

**Figure 8:**
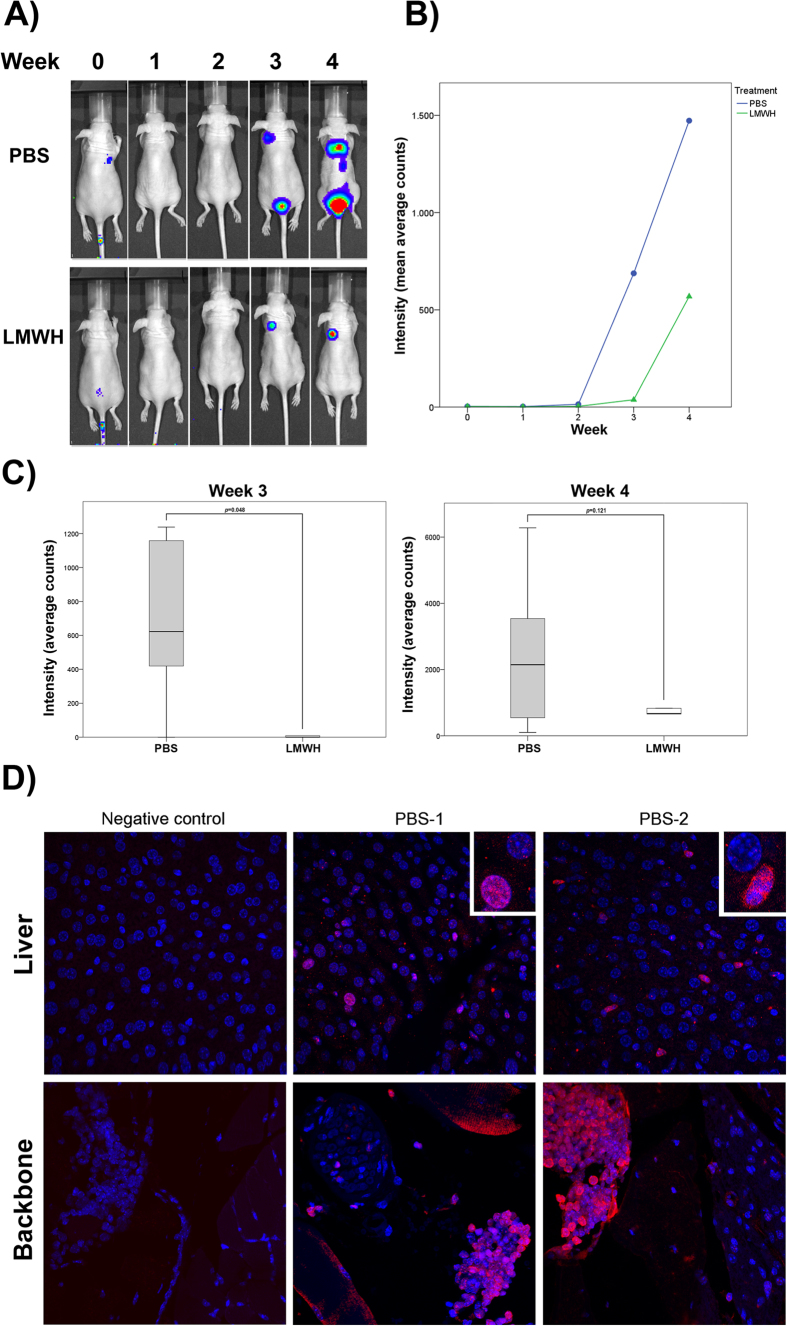
Low molecular weight heparin reduces metastatic potential *in vivo*. (**A**) Representative whole body bioluminescence images of mice showing metastatic colonies at week 0, 1, 2, 3 and 4 after injection into the tail vein of HT-29 cells and previous treatment with 100 U Low molecular weight heparin (LMWH) or vehicle (PBS). (**B**) Bioluminescence imaging assay of tumors in mice at week 0, 1, 2, 3 and 4 post treatment and injection of cells. Mean bioluminescence counts ± SEM (n = 5). (**C**) Quantification of metastasis as assessed by bioluminescence measurements at week 3 and week 4 from the tail vein injection of HT-29 cells after previous treatment of mice with PBS or LMWH. Statistical analysis was carried out with the Student’s t-test. (**D**) Immunological detection of luciferase protein expression in liver and backbone by confocal microscopy. Histological sections of two control mice treated with PBS showing HT29-CMV-luciferase cells invasion. Signal of secondary antibody (negative control) is also shown (left panel). Scale bar: 100 μm

Since the *in vitro* results showed that antithrombin may have a role in the inhibition of the migration and invasion while the effect on proliferation was negligible, we studied the effect of LMWH on experimental metastasis using an *in vivo* animal model. HT29 cells expressing luciferase were injected into the tail vein of athymic nude mice. In a group of animals, a single dose of LMWH was intraperitoneally inoculated 30 min before tumor cell injection, while a second group of mice (control) received the vehicle (PBS). In both groups, only 5 out of 10 mice developed detectable metastases the third week post-injection, and the number of animals with metastases increased to 6 the fourth week (Supplementary Fig. S9). Nevertheless, metastatic lesions of mice treated with LMWH had a reduced area compared to control mice, as shown by measuring the intensity of luminescence (Fig. 8A–C). These differences were statistically significant at week 3 post-injection, but although a decreased intensity of luminescence was still observed in the LMWH group significance was lost at week 4 (Fig. 8C). The presence of metastatic cells in liver and backbone was detected by luciferase immunostaining of histological sections (Fig. 8D). The staining was only detected in sections corresponding to control mice, maybe due to undetectable micrometastasis in the LMWH group.

## Discussion

Enteropeptidase is a serine protease that belongs to the type II transmembrane serine protease superfamily. It has been reported that the members of this superfamily are involved in the progression of some tumors^21^,^22^,^23^, although the physiological function of enteropeptidase is the conversion of zymogens into active proteases for food digestion^24^. Nevertheless, enteropeptidase has never been implicated in tumorigenic processes. Here, we confirm the expression of enteropeptidase in cell lines from colorectal, glioblastoma and lung cancer origins and, to our knowledge, demonstrate for the first time the direct role of enteropeptidase in tumor migration and matrix degradation. Our results show that the addition of antithrombin and especially antithrombin activated by LMWH, delayed cell migration and reduced matrix degradation of cells expressing enteropeptidase. These two processes (cell migration and matrix degradation) are key steps in facilitating tumor invasion and metastasis, and appear to be controlled by enteropeptidase as demonstrated by silencing its expression. In fact, treatment with LMWH was able to reduce the metastatic ability of tumor cells expressing enteropeptidase in an *in vivo* animal model. It has been shown that enteropeptidase may be inhibited by another serpin, PCI^12^. However, it could not be demonstrated that this inhibition occurs *in vivo* because the two proteins (PCI and enteropeptidase) did not co-localize in the tissue. Our results show that antithrombin might function as an *in vivo* inhibitor of enteropeptidase in pathological conditions, in which the two proteins may be in contact, thus increasing the likelihood of the interaction, although there should be heparin present. Nevertheless, as with antithrombin, PCI could also extravasate and inhibit enteropeptidase in tumors, being both serpins regulators of enteropeptidase function.

The inhibition of enteropeptidase by antithrombin appears to be accelerated by heparin. The fact that pentasaccharide and LMWH provoked a stronger inhibition of enteropeptidase by antithrombin than did unfractionated heparins indicated that this process depends on the allosteric activation of antithrombin and that long chain heparins may interfere with the inhibition of enteropeptidase. Moreover, the inhibition of enteropeptidase requires the two functional domains of antithrombin (the RCL and the heparin binding domain)^13^,^14^,^15^,^16^. However, the results obtained when cells were incubated with antithrombin and the different heparins, indicate that, in this process, LMWH is more efficient.

The anti-tumor effect of LMWH has been extensively evaluated. Clinical trials have demonstrated its effect on increasing the survival of cancer patients with and without venous thrombotic disease, independently of its effect on coagulation^25^,^26^,^27^,^28^. However, additional trials are required to define the tumor types, disease stages and dosage schedules that provide the greatest survival benefit^29^. There are many biological roles of LMWH; it can block several crucial events during the tumorigenic process^30^,^31^. A critical event in invasion and metastasis is the degradation of extracellular matrix components, such as collagen, laminin, fibronectin and proteoglycans^32^. Tumor cells are able to break down the extracellular matrix by secretion of metalloproteases, serine proteases, cysteine proteases and endoglycosidases, such as heparanase^33^, whose function may be impaired by LMWH^34^. However, some of the anti-tumor effects of heparins, the main cofactor of antithrombin, might also be mediated by the inhibitory function of antithrombin. In fact, the inhibitory effect of antithrombin on matriptase, a transmembrane serine protease involved in matrix remodeling, has been previously reported^35^. Although proliferation of cells expressing enteropeptidase is not affected by treatment with antithrombin, cell migration and matrix degradation (key steps for tumor invasion) were significantly inhibited. Moreover, the reduction in the migration and invasion of the cells was exacerbated when they were incubated with activated antithrombin. These events may indicate that some of the anti-tumor effects attributed to heparins might be mediated by the function of antithrombin. Our *in vivo* results demonstrated that treatment with LMWH, previously to the injection of the tumor cells expressing enteropeptidase into the tail vein of mice, reduced the extent of metastatic lesions in comparison to those treated with vehicle. However, metastatic colonies were able to grow also in mice treated with LMWH. This is in concordance with the ABEL study^36^, in which authors concluded that prophylactic treatment with LMWH improved overall survival and disease free survival only in patients with limited-stage small cell lung cancer. Recently, it has been published that over-expression of the serpin member heparin co-factor II (HCII) in non-small cell lung cancer is associated with increased metastasis and that binding of heparin blocks the pro-metastatic action of HCII^37^. Therefore, the action of LMWH in impairing tumor cell migration/invasion and metastasis, as shown in this paper, might be mediated at least in part by HCII or by the effect of LMWH on other proteins involved in tumor cell growth and metastasis, such as the receptor for advanced glycation end-products axis or on the up-regulation of PI3K by HCII^37^,^38^. Nevertheless, we speculate that patients with tumors expressing enteropeptidase may benefit from the treatment with heparins, and we cannot exclude that antithrombin might also inhibit other proteases involved in tumor progression.

In this work, we demonstrated a unique property of the inhibition of enteropeptidase by antithrombin. The interaction between enteropeptidase and antithrombin did not involve the formation of covalent complexes. An unexpected consequence of enteropeptidase inhibition by antithrombin was angiogenesis inhibition. Our results showed that, after interaction with enteropeptidase, antithrombin remained in a cleaved conformation. This result is especially relevant because an anti-angiogenic role has been attributed to the cleaved protein^5^, and we showed that antithrombin, after incubation with enteropeptidase, inhibited vessel formation of endothelial cells even in co-culture with tumor cells. The enteropeptidase-dependent antiangiogenic effects of antithrombin might be especially relevant in the setting of the tumor models glioblastoma, lung cancer and colorectal cancer. All of these are prevalent tumors and show prominent angiogenic activation; consequently, they are usually treated with antiangiogenic drugs such as bevacizumab. The antiangiogenic therapeutic strategy leads to consistent improvements in progression free and overall survival in patients with advanced disease bearing any of these tumors, in clear contrast with the effects of the same drug in other neoplasms^39^,^40^,^41^. No data are available on enteropeptidase expression in these neoplasms in a clinical setting, but our results warrant further work to delineate its potential in tumor progression and, especially, to better define the role of antithrombin, enteropeptidase and heparin in their angiogenic behavior.

In summary, to our knowledge, here we provide the first demonstration that antithrombin may have an anti-tumor effect through a double mechanism, inhibition of a protease involved in tumor migration and invasion and generation of anti-angiogenic cleaved antithrombin.

## Materials and Methods

### Cell lines

MDA-MB-231, MCF7, KATO III, and Caco-2 were obtained from the Service of Cell Culture, Investigation Support Service (S.A.I, University of Murcia). U-87 MG and A549 were kind gifts from Dr. M.I. Martínez-Lacaci (IMIB-Arrixaca, Murcia, Spain) and Dr. P. Martín-Duque (Francisco de Vitoria University, Madrid, Spain), respectively. The human EA. hy926 endothelial cell line was a kind gift from Dr. C.-J. S. Edgell (University of North Carolina, USA). All cell lines were cultured following the ATCC animal cell culture guide and using recommended culture mediums. All cell lines were tested for mycoplasma contamination using Venor®GeM Classic Mycoplasma PCR Detection Kit (Minerva Biolabs, Berlin, Germany), and authentication was determined by Short tandem repeat profiling^42^.

### qRT-PCR

The expression of *TMPRSS15* gene in all cell lines was determined by qRT-PCR using SYBR Premix Ex Taq II (Takara) in a LightCycler480 (Roche), following the manufacturer’s indications. The primers used for the amplification of *TMPRSS15* gene and beta-actin, as a reference gene, are described in the Supplementary Information section.

### Incubation with antithrombin and enteropeptidase solubilization of cell lines

Antithrombin (150 ng/μL) and/or heparin (200 U/mL) were added to the cells (HT-29, U-87 MG and A549), which were then incubated at 37 °C. After 1 hour of incubation, medium was withdrawn, and cells were washed three times with PBS.

The cells were detached by adding a lysis buffer (20 mM Tris HCl, 1% Triton X-100, pH 7.5) and using scrapers. Then, cells were centrifuged at 13684 *g* for 10 min at 4 °C. The supernatant was recovered and then used for the determination of enteropeptidase activity. To determine enteropeptidase activity, Z-Lys-SBzl was used at 200 μM in TCNB buffer (50 mM Tris, 0.15 M NaCl, 10 mM CaCl2, and 0.05% Brij-35,pH 7.5) containing 200 mM Ellman’s reagent (5,5′-dithiobis-(2-nitrobenzoic acid)). The absorption was recorded at 405 nm for 60 min to assess the activity of enteropeptidase.

### *In vitro* inhibition of enteropeptidase by antithrombin

Formation of covalent complexes between antithrombin and enteropeptidase (Sigma, Madrid, Spain) was evaluated by adding an excess of enteropeptidase (150 μM) to 5 μM of antithrombin and incubating the complex at different times (0, 15, 30, 45 and 60 min) at 37 °C. Complexes were evaluated by 8% SDS-PAGE under reducing and non-reducing conditions, as described elsewhere^43^.

To evaluate which functional domains of antithrombin are required for the inhibition of enteropeptidase, two different antithrombin mutants were used, and enteropeptidase activity was measured (antithrombin London and antithrombin Toyama). Mutagenesis of plasmid containing cDNA coding for antithrombin, transfection of HEK-EBNA cells and purification of variants were carried out as previously described^13^,^15^.

### Edman sequencing

Edman sequencing of the antithrombin cleaved by enteropeptidase was performed under non-reducing conditions with Applied Biosystems Procise 494 equipment (Foster City, CA, USA). Because of the presence of a C-terminal disulfide bond between Cys247 and Cys430, cleavage at the reactive center loop of antithrombin is detected as the cleaved peptide remains bound^17^,^18^.

### Effect of antithrombin on vessel formation of endothelial cells EA. hy926 and in co-culture with U-87 MG cells

Cells were added to a 96-well plate (15000 cells in 100 μL of medium deprived of fetal bovine serum to each well) previously treated with 9 μL of Matrigel. The conditions studied were as follows: 1) 10 μM D,L-sulforaphane; 2) 2.5 μM antithrombin, 2.5 μM enteropeptidase, and 200 U/mL LMWH, previously incubated together at 37 °C for 24 hours; 3) 2.5 μM antithrombin; 4) 2.5 μM enteropeptidase; 5) 200 U/mL LMWH. Each condition was assayed in eight different wells. D,L-sulforaphane was used as a positive control in this assay^44^. Co-culture of EA. hy926 and U-87 MG cells was also assayed to determine the effect on vessel formation. As described, 15000 cells from each of the cell lines were added to the 96-well plate under the same conditions as described above. The conditions studied were as follows: 1) 10 μM D,L-sulforaphane; 2) 2.5 μM antithrombin, 2,5 μM enteropeptidase, and 200 U/mL LMWH, previously incubated together at 37 °C for 24 hours; 3) 2.5 μM antithrombin; and 4) 200 U/mL LMWH. Each condition was assayed in eight different wells.

The second condition of both assays generated cleavage of antithrombin by enteropeptidase, as verified by 8% SDS-PAGE under non-reducing conditions and silver staining. The lengths of endothelial tubes formed in each well was quantified from captured images using Angiogenesis Analyzer for ImageJ when endothelial cells were assayed alone and manually using segmental lines and setting scale in ImageJ when co-culture was analyzed. Statistical analysis was performed with a Mann-Whitney U test in IBM SPSS Statistics 21 software.

### Silencing of TMPRSS15

U-87 MG cells were grown in a humidified atmosphere with a 5% CO_2_ concentration. ON-TARGETplus SMARTpool siRNAs against *TMPRSS15* and control siRNA were obtained from Dharmacon (GE Dharmacon, Barcelona, Spain). siRNA transfections were performed using PepMute transfection reagent (SignaGen, MD, USA). Silencing rates were approximately 80% after two days of transfection (Supplementary Fig. S10).

### Proliferation and wound healing assays

After 24 hours, cell proliferation was assessed using XTT according to the manufacturer’s protocol (ATCC, Manassas, VA, USA). Six replicates for each condition were examined. The three following conditions were studied in comparison with cells without any treatment: 1) 2.5 μM antithrombin, 2) 200 U/mL LMWH, and 3) 2.5 μM antithrombin and 200 U/mL LMWH. Absorbance values at 450 nm (corrected at 690 nm) were measured using a microplate spectrophotometer.

For the wound healing assay, cells were cultured as confluent monolayers, synchronized in 1% FBS for 24 h, and wounded by removing a 300–500 μm-wide strip of cells across the well with a standard 200 μl pipette tip. Wounded monolayers were washed twice to remove non-adherent cells. The following three conditions were studied in comparison to cells without any treatment: 1) 2.5 μM antithrombin, 2) 200 U/mL LMWH, 3) 2.5 μM antithrombin and 200 U/mL LMWH. The same experiment was carried out with *TMPRSS15*-silenced U-87 MG cells in the absence and presence of the combination of 2.5 μM antithrombin and 200 U/mL LMWH. Wound healing was quantified using ImageJ software as the median percentage of the remaining cell-free area compared to the area of the initial wound. Statistical comparisons were performed using the Mann–Whitney U test.

### Matrigel invasion assay and invadopodia degradation of gelatin coated coverslips

Matrigel invasion assays were performed at 37 °C for 6 hours using 24-well transwell inserts coated with Matrigel (BD Biosciences, Madrid, Spain). U-87MG and A549 cells 200000 suspended in 400 μL serum-free medium were seeded into the upper chamber and 500 μL serum-supplemented medium was added in the lower chamber. The following three treatments were analyzed in comparison to cells without treatment: 1) 2.5 μM antithrombin, 2) 200 U/mL LMWH, and 3) 2.5 μM antithrombin and 200 U/mL LMWH. Cells that migrated and invaded through the membrane were counted after fixing and staining with crystal violet. Images were analyzed with Fiji software and processed with Adobe Photoshop. Statistical significance was analyzed by the Mann–Whitney U test.

For evaluation of invadopodia degradation, gelatin matrix was prepared by mixing 0.2% gelatin and Rhodamine (Invitrogen, Life Technologies, Madrid, Spain) at a 1:55 ratio in 61 mM NaCl, 50 mM sodium borohydrate and dialyzed overnight in PBS. Coverslips were first coated with this mix and then fixed with 0.5% glutaraldehyde for 15 min. After coating, coverslips were washed with PBS. For the degradation assay, U-87 MG cell suspensions were plated on top of these coated coverslips for 24 hours in the presence of 25 ng/mL hGF (Sigma-Aldrich, Madrid, Spain). The same three conditions as above were studied in comparison to cells without any treatment, as follows: 1) 2.5 μM antithrombin, 2) 200 U/mL LMWH, and 3) 2.5 μM antithrombin and 200 U/mL LMWH. The same experiment was carried out with silenced TMPRSS15 cells in the absence or presence of the combination of 2.5 μM antithrombin and 200 U/mL LMWH. Immunofluorescence analysis was carried out after fixing the cells with 3.7% formaldehyde and incubation with phalloidin, Alexa Fluor 488 and DAPI^45^. Images were taken with a Nikon 90i microscope at 60×, analyzed with Fiji software and processed with Adobe Photoshop^45^. Cells that degraded gelatin were scored as positive, and more than 100 random selected cells were quantified. The percentage of cells with invadopodia-mediated degradation was plotted and statistical significance was analyzed by the Mann–Whitney U test. Global association was also statistically analyzed by Kruskal-Wallis test.

### Carcinoma cell xenografts in nude mice and immunodetection of antithrombin and enteropeptidase in tumors

Described in detail in Supplementary Materials and Methods.

### Experimental metastasis in nude mice and treatment with heparin

Animals were kept in ventilated rooms under lighting (12-h light, 12-h dark cycle) and temperature controlled conditions, and allowed feed and water *ad libitum*. All experimental procedures were conducted in compliance with 2010/63/UE European guidelines.

5 × 10^5^ Colon cancer cells (HT-29), infected with lentiviral expression particles for luciferase (CMV-Luciferase firefly, from Amsbio Abingdon, UK), were injected into the tail vein of 6-weeks old *nu/nu* mice 30 minutes after intraperitoneal injection of 100 units of LMWH (n = 10), or control PBS (n = 10), under anesthesia (Isofluorane). Each mouse was imaged periodically, using an IVIS Lumina 2 imaging system (Xenogen, Alameda, CA) after retro-orbital injection of 3 mg luciferin (Goldbio., St. Loius, MO, USA) in 100 μl of DPBS, under anesthesia, using 5 min, 2 min and 30 sec exposure time. After 4 weeks, mice were euthanized and pieces of liver and backbone were excised.

### Immunohistochemical analysis

For immunolocalization of luciferase in histological sections, pieces of liver and backbone were fixed in pH 7.0 buffered 3.7% formaldehyde and processed for histology. Hydrated histological sections were stained with goat pAb against luciferase (Novus Biologicals, Littleton, CO), after permeabilization in 0.1% Triton X-100, antigen retrieval in citrate buffer (0,01 M, pH6), and blocking in 0.5% BSA. The immunofluorescent signal of liver sections was revealed using donkey anti goat IgG secondary antibody, Alexa Fluor 546-coupled. The primary antibody signal in backbone sections was amplified using donkey anti goat IgG, HRP coupled, secondary antibody and TSA Plus Cyanine 3 (Perkin Elmer, Massachusetts, USA), following the manufacturer’s instructions. In both cases, cells nuclei were counterstained with DAPI. Histological sections were also stained with standard haematoxylin-eosin for routine evaluation of tissue morphology. Confocal laser-scanning microscopy was performed in a Leica TCS-SP2 microscope (Leica Microsystems, Heidelberg, Germany). Images were acquired using a 63× (NA 1.32) oil-immersion objective and assembled using Leica Confocal Software 2.0.

### Ethic Statement

The ethical committee “CEIC Hospital General Universitario José María Morales Meseguer” approved the experimental protocols included in this paper.

## Additional Information

**How to cite this article**: Luengo-Gil, G. *et al*. Antithrombin controls tumor migration, invasion and angiogenesis by inhibition of enteropeptidase. *Sci. Rep*. **6**, 27544; doi: 10.1038/srep27544 (2016).

## Supplementary Material

Supplementary Information

## Figures and Tables

**Table 1:** Enteropeptidase activity using its chromogenic substrate after solubilization from U-87 MG cells.

	Vmax (mOD/min)
–	5.29 ± 0.2
LMWH	5.27 ± 0.3
AT	1.80 ± 0.3
AT + LMWH	0.20 ± 0.1
AT London + LMWH	5.06 ± 0.2
AT Toyama + LMWH	2.94 ± 0.2

Enteropeptidase activity was evaluated after incubation of cells in the absence (−) or presence of antithrombin (AT), AT activated by low molecular weight heparin (LMWH), AT London (ΔR393) in presence of LMWH, or AT Toyama (R47C) in presence of LMWH. The values are represented as the mean of three different experiments.
