# Supplementary material for: Antithrombin controls tumor migration, invasion and angiogenesis by inhibition of enteropeptidase
Source: Sci Rep. 2016 Jun 8;6:27544. doi: 10.1038/srep27544 (PMC4897635; doi:10.1038/srep27544)
Supplement: Supplementary Information [file srep27544-s1.pdf]

SUPPLEMENTARY INFORMATION

Title: Antithrombin controls tumor migration, invasion and angiogenesis by inhibition of enteropeptidase

Ginés Luengo-Gil*[1], María Inmaculada Calvo*[2], Ester Martín-Villar[2], Sonia Águila[1], Nataliya Bohdan[1], Ana I. Antón[1], Salvador Espín[1], Francisco Ayala[1], Vicente Vicente[1], Javier Corral[1], Miguel Quintanilla[2], Irene Martínez-Martínez[1]

[1]Servicio de Hematología y Oncología Médica, Hospital Universitario Morales Meseguer, Centro Regional de Hemodonación, Universidad de Murcia, IMIB-Arrixaca, Murcia.

[2]Instituto de Investigaciones Biomédicas Alberto Sols, CSIC-UAM, Madrid.

Corresponding author's contact information:

Dr. Irene Martínez-Martínez
Centro Regional de Hemodonación
Ronda de Garay S/N, Murcia 30003, Spain
Tel: +34968341990
Fax: +34968261914
E-mail: immlgi@um.es

* These authors contributed equally to this article

Detailed methods

Materials

Pentasaccharide (Fondaparinux; Arixtra®) was from Sanofi-Synthelabo (Paris, France), and LMWH (Bemiparin) or UFH were from Rovi (Madrid, Spain).

Culture media were obtained from Gibco (Fisher Scientific, Madrid, Spain). NP-40, recombinant human D,L-sulforaphane, recombinant active enteropeptidase, glutaraldehyde and rhodamine were purchased from Sigma (Madrid, Spain). Matrigel™ Basement Membrane Matrix was obtained from BD Biosciences (Madrid, Spain). Antithrombin was purified from plasma as described elsewhere (1).

qRT-PCR

Transcript relative quantification of TMPRSS15 was performed by qRT-PCR using SYBR Premix Ex Taq II (Takara) and the primers 5'CTAGGCCTGCATATGAAATC3' (forward) and 5'CTGTGTAATTCACTTTAAATTCC3' (reverse). Expression of beta-actin (*ACTB*) was employed as endogenous reference control, using the primers 5' TGACCCAGATCATGTTTGAGA3' (forward) and 5'TAGCACAGCCTGGATAGCAA3' (reverse). The PCRs were performed using a LightCycler 480 system (Roche). Expression analysis was performed in duplicate for each sample. The fold difference for each sample was obtained using the second derivative Ct method (2).

Carcinoma cell xenografts in nude mice and immunodetection of antithrombin and enteropeptidase in tumors

Animals were kept in ventilated rooms under lighting (12-h light, 12-h dark cycle) and temperature controlled conditions, and allowed feed and water *ad libitum*. All experimental procedures were conducted in compliance with 2010/63/UE European guidelines.

HT-29, U-87 MG and A549 were injected ($5x10^6$ cells) subcutaneously into the flanks of 6-week old female nude mice. Mice were euthanized and tumors were surgically removed when their diameters reached over 1 cm. Only HT-29 and A549 cells were tumorigenic.

For immunoblotting, excised tumors were homogenized at 4°C in NT lysis buffer (50 mM Tris-HCl, pH 7.4, 100 mM NaCl, 5 mM $MgCl_2$, 5 mM $CaCl_2$, 1% NP-40, 1% Triton X-100, 2 mM phenylmethylsulfonyl fluoride, 20 μg/ml aprotinin, and 1 mM sodium orthovanadate). For immunofluorescence analysis, sections from paraffin-embedded tumors were fixed and subjected to heat-induced antigen retrieval before exposure to primary antibodies. Polyclonal antibodies recognizing antithrombin (Sigma-Aldrich Cat# A9522 RRID:AB_258455) and anti-enterokinase LC (L-13) (Santa Cruz Biotechnology Cat# sc-51283 RRID:AB_2253102) were used. Secondary anti-rabbit and anti-goat Alexa Fluor 594 and Alexa Fluor 488 antibodies were from Invitrogen (Life Technologies Cat# A21207 RRID: AB_10049744 and Life Technologies Cat# A11055 RRID:AB_10564074, respectively). The covers were then washed three times with PBS and twice with distilled H_2O before being mounted in Vectashield mounting medium (Vector Laboratories, UK). Confocal laser-scanning microscopy was performed in a Leica TCS-SP2 microscope (Leica Microsystems, Heidelberg, Germany). Images were acquired using a 63× (NA 1.32) oil-immersion objective and

assembled using Leica Confocal Software 2.0. Secondary antibodies were also incubated in the absence of primary antibodies to detect unspecific immunostaining.

References

1. Mushunje, A., Evans, G., Brennan, S.O., Carrell, R.W. & Zhou, A. Latent antithrombin and its detection, formation and turnover in the circulation. *J. Thromb. Haemost.* **2**, 2170-2177 (2004).
2. Schmittgen, T. D., & Livak, K. J. Analyzing real-time PCR data by the comparative CT method. *Nature Protocols*, ***3***, 1101-1108 (2008).

Supplementary figure legends

Supplementary Fig. S1. *TMPRSS15* gene and protein expression in A549, U-87 MG and HT-29 cells. A) Amplification curves for *TMPRSS15* gene by RT-qPCR. The target-specific fluorescence signal of SYBR Green fluorescence emission (detection range 515–545 nm) is plotted against the number of PCR cycles. RT-PCR experiments were carried out in duplicate. The threshold level is given by green horizontal lines. B) Electrophoretic mobility of enteropeptidase. SDS-PAGE was run under reducing conditions and enteropeptidase was detected by western blot and immunostaining.

Supplementary Fig. S2. Electrophoretic mobility of antithrombin in the supernatant of cells after incubation for 1 hour. SDS-PAGE was run under non-reducing conditions and antithrombin was detected by western blot and immunostaining. Supernatant of MDA-MB-231 cells was also loaded as a control of

cells with low expression of enteropeptidase. AT-T: antithrombin cleaved by thrombin. ATc: control purified antithrombin.

Supplementary Fig. S3. Electrophoretic mobility of cleaved antithrombin by enteropeptidase. SDS-PAGE was run under non-reducing conditions and proteins were detected by silver staining. AT: antithrombin; EP: enteropeptidase; LMWH: low molecular weight heparin.

Supplementary Fig. S4. Enteropeptidase protein expression in EA.hy 926. SDS-PAGE was run under reducing conditions and proteins were detected by immunodetection. U-87 MG, HT-29 and A549 cells were also run as positive controls of enteropeptidase expression. Beta-actin expression was used as a loading control.

Supplementary Fig. S5. Cell proliferation assay of U-87 MG, A549 and HT-29 cells. Cells were grow in PBS or in presence of low molecular weight heparin (LMWH), antithrombin (AT) or low molecular weight heparin and AT in combination (AT+LMWH) and proliferation was determined using a XTT kit (ATCC, Manassas, VA, USA) at 24 hours. Each histogram represents the mean ± SD of three experiments.

Supplementary Fig. S6. Effect of antithrombin and heparin on cell migration of HT-29 and A549 cells. Wound healing was evaluated after incubation of cells for 24 hours. A) Microscope images of HT-29 cells 0 and 24 hours after the wound with the pipette tip. B) Microscope images of A549 cells 24 hours after the wound. The conditions assayed were: no treatment, incubation with low molecular weight heparin (LMWH), antithrombin (AT) or antithrombin and LMWH in combination (AT-

LMWH). C) Representation of percentage of wound confluence under the different conditions. Each condition was evaluated in triplicates and five different images were processed for each different assay; *: $p<0.05$; **: $p<0.01$. Images were recorded by a Leica microscope at 5×, and Image-J was used to analyze migration. Mann-Whitney U test was used to determine the significance.

Supplementary Fig. S7. Effect of antithrombin and heparin on invasion of A-549 cells by a transwell assay. Cell invasion was evaluated after incubation of cells for 9 hours under the following conditions: no treatment, incubation with low molecular weight heparin (LMWH), antithrombin (AT) or incubation with antithrombin and LMWH in combination (AT-LMWH). A) Microscope images of cells invaded after 9 hours of incubation. B) Percentage of cell invasion under the different conditions. Each condition was evaluated in triplicate, and three different images were processed for each different assay; *: $p<0.05$; **: $p<0.01$. Images were recorded with a Leica microscope at 5×, and Image J was used to analyze the invaded area. Statistical analysis was carried out with a Mann-Whitney U test.

Supplementary Fig. S8. Antithrombin and enteropeptidase immunodetection in human xenograft tumors surgically removed from nude mice. HT-29 and A549 tumor cells were subcutaneously injected in the two flanks (T1 and T2) of nude mice and removed when they reached around 1 cm diameter. Immunostaining by western-blot was performed with the same primary antibodies used for the immunohistofluorescence assay. T1 and T2 represent different xenograft tumors Alpha-tubulin was detected as loading control.

Supplementary Fig. S9. Effect of low molecular weight heparin on the metastatic potential *in vivo*. Bioluminescence images of mice showing metastatic colonies at week 3 and 4 after injection into the tail vein of HT-29 cells. Mice were previously treated with 100 U Low molecular weight heparin (LMWH) or vehicle (PBS). Only those mice with detectable metastasis are shown.

Supplementary Fig. S10. *TMPRSS15* gene silencing efficiency in U-87 MG cells. A) Quantitative real time PCR data showing a >80% suppression of *TMPRSS15* gene expression. Cells were transfected with 5 nM ON-TARGETplus SMARTpool siRNAs against *TMPRSS15*, and control siRNA. Relative expression of mRNA was calculated using the comparative CT method 48 hours after transfection. Experiments were performed in triplicates. B) Enteropeptidase expression in silenced cells. SDS-PAGE was run under reducing conditions. Beta-actin expression was used as a loading control.

Supplementary Tables

Supplementary Table S1. Tumorigenicity of human U-87 MG, A549 and HT-29 cell xenografts in *nu/nu* mice.

	U-87 MG	HT-29	A549
**Days to observe tumors 0.5 cm^2 of size**	N.A	4-6	22-25
**Number of tumors per injection sites (%)**	0	100	100
**Number of mice**	7	7	7

N.A. (not applicated)

Supplementary Table S2. Statistical analysis of vessel formation by the co-culture of EA. hy926 and U-87 MG cells under different conditions.

Comparisons*	*p*
All conditions	0.00009†
DL-Sulforaphane	0.008‡
DL-Sulforaphane Vs AT	0.008‡
DL-Sulforaphane Vs LMWH	0.011‡
DL-Sulforaphane Vs AT-EP-LMWH	NS‡
No treatment Vs AT	0.085‡
No treatment Vs LMWH	0.203‡
No treatment Vs AT- EP-LMWH	0.001‡
AT Vs LMWH	0.908‡
AT Vs AT-EP-LMWH	0.001‡
LMWH Vs AT-EP-LMWH	0.001‡

* 8 replicates for each condition
† Kruskal-Wallis test
‡ Mann-Whitney U test
NS: Non-Significant

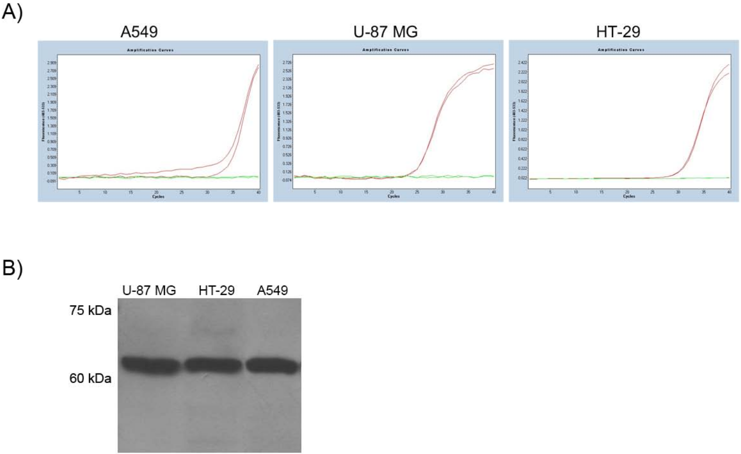
A)
A549
U-87 MG
HT-29
Amplification Curves
Cycles
B)
U-87 MG
HT-29
A549
75 kDa
60 kDa

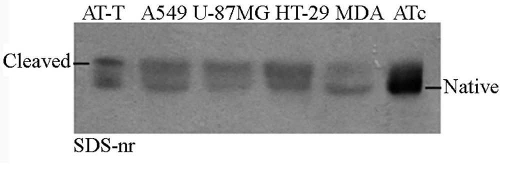
AT-T
A549
U-87MG
HT-29
MDA
ATc
Cleaved
Native
SDS-nr

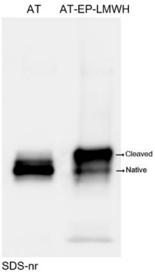
AT
AT-EP-LMWH
Cleaved
Native
SDS-nr

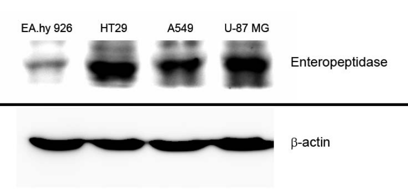
EA.hy 926
HT29
A549
U-87 MG
Enteropeptidase
β-actin

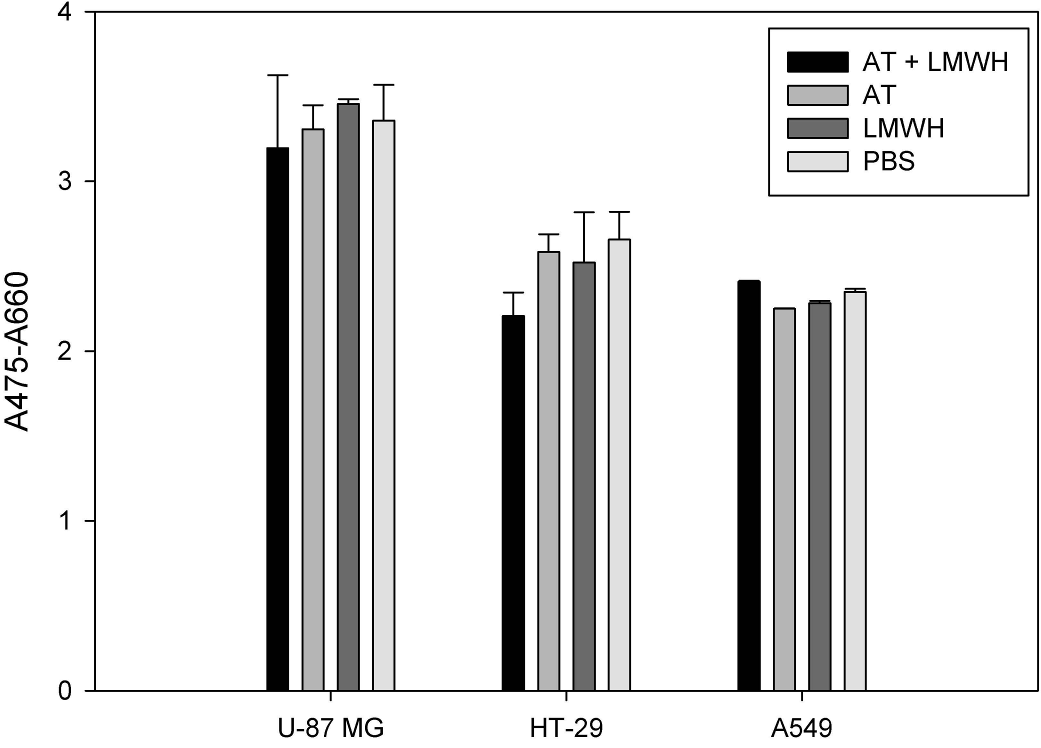

4
3
2
1
0
A475-A660
AT + LMWH
AT
LMWH
PBS
U-87 MG
HT-29
A549

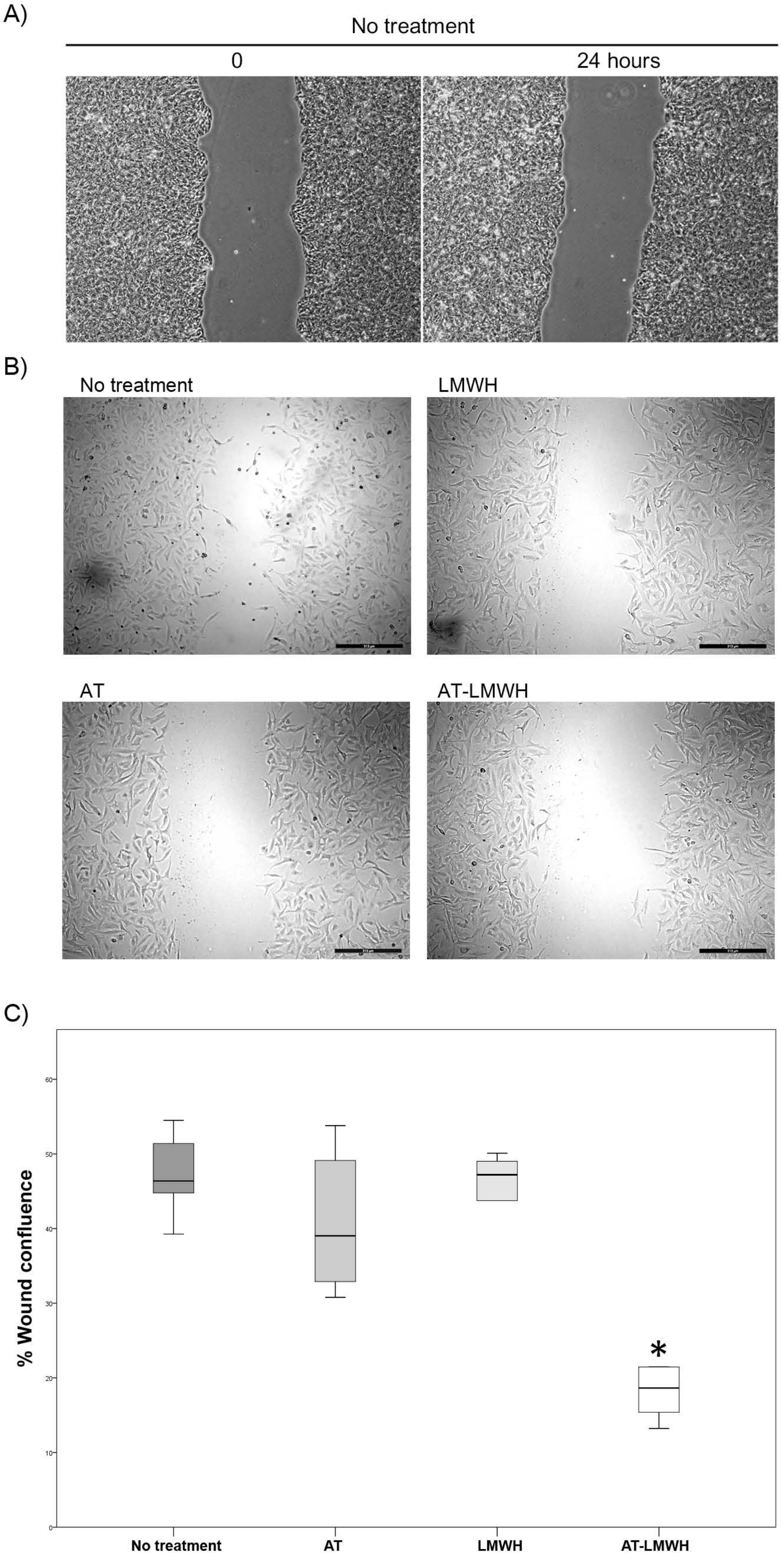
A)
No treatment
0
24 hours
B)
No treatment
LMWH
AT
AT-LMWH
C)
% Wound confluence
60
50
40
30
20
10
0
*
No treatment
AT
LMWH
AT-LMWH

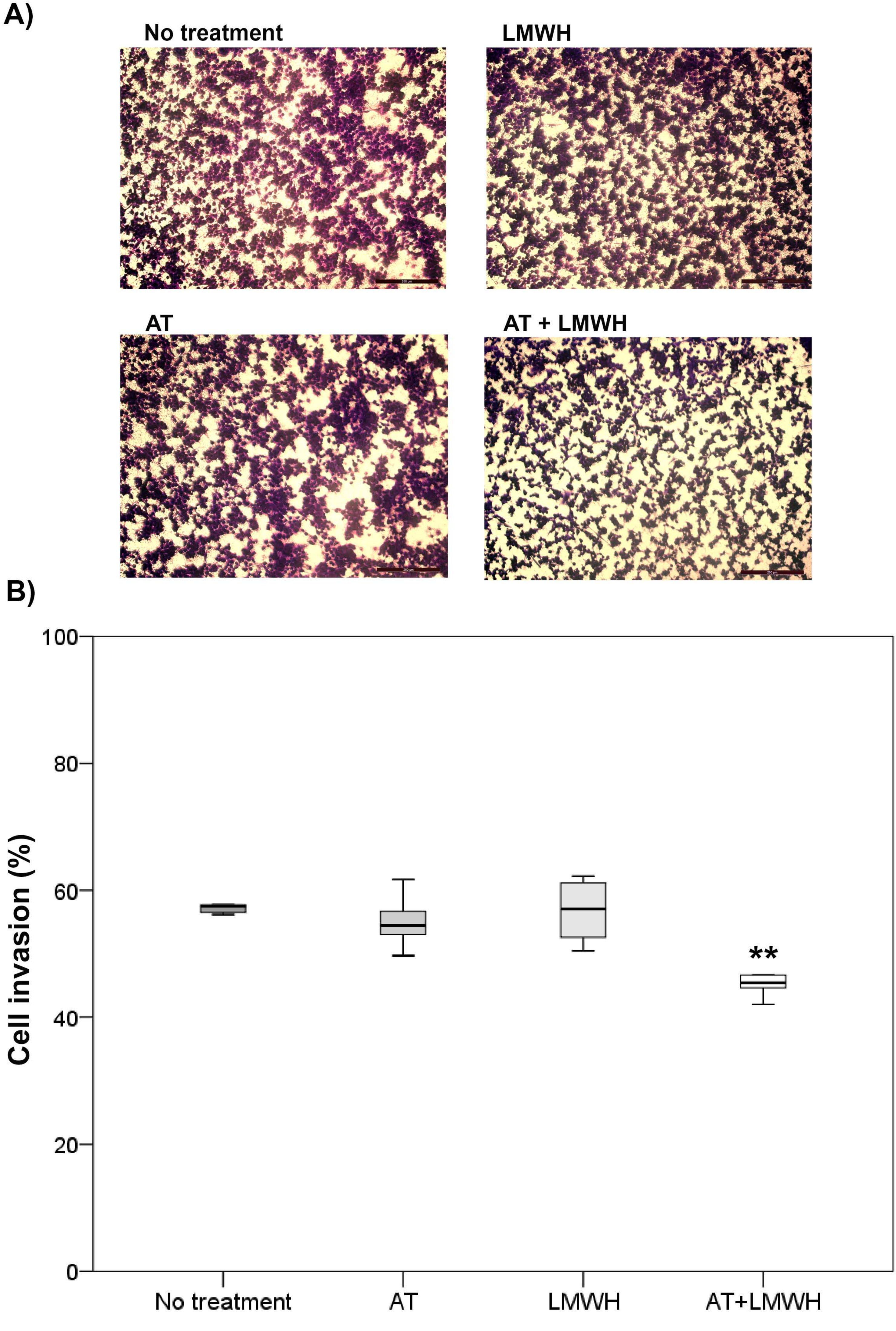
A)
No treatment
LMWH
AT
AT + LMWH
B)
Cell invasion (%)
100
80
60
40
20
0
**
No treatment
AT
LMWH
AT+LMWH

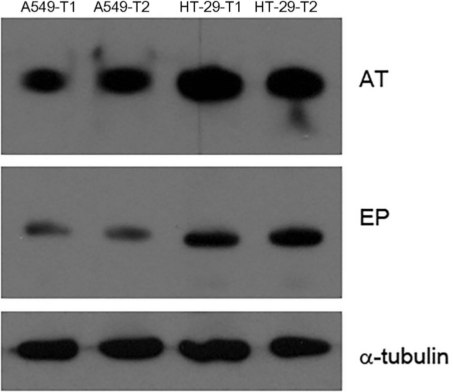

A549-T1
A549-T2
HT-29-T1
HT-29-T2
AT
EP
α-tubulin

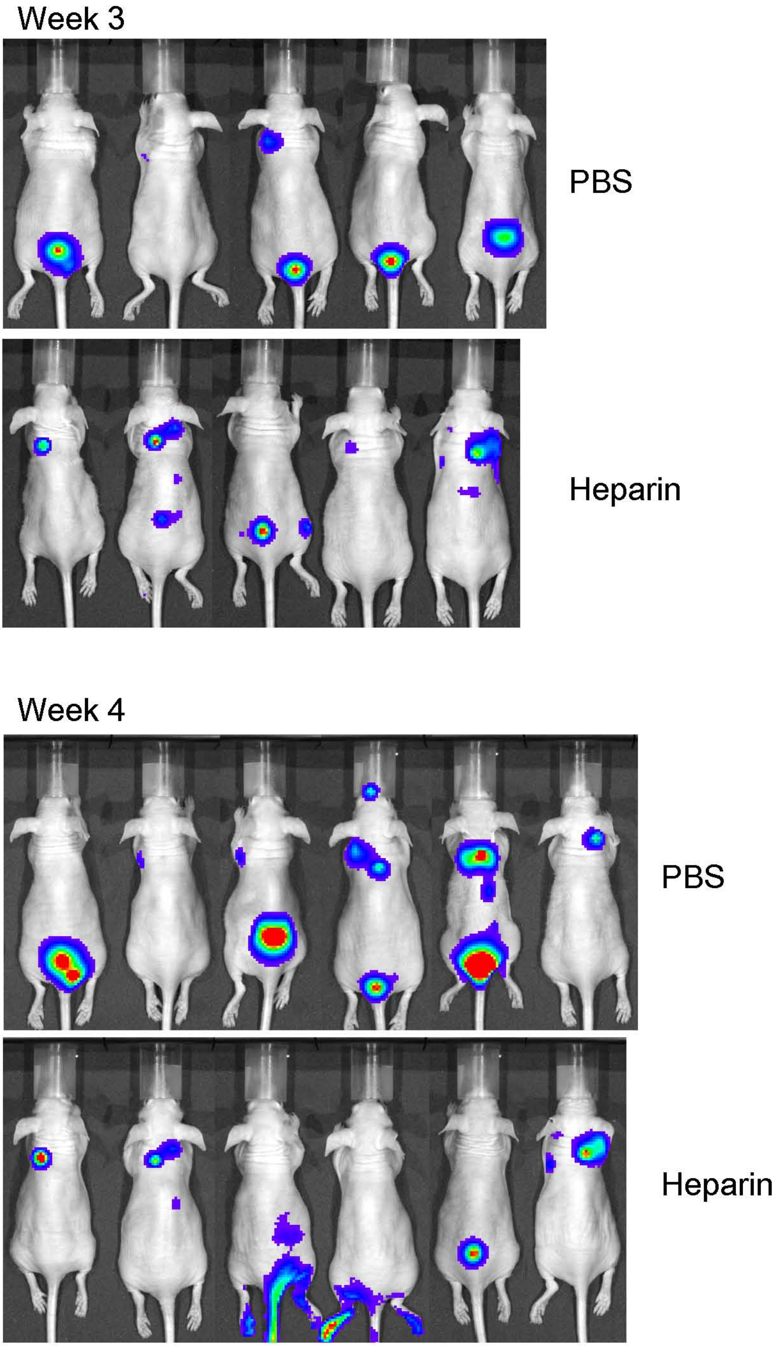
Week 3
PBS
Heparin
Week 4
PBS
Heparin

A)

B)

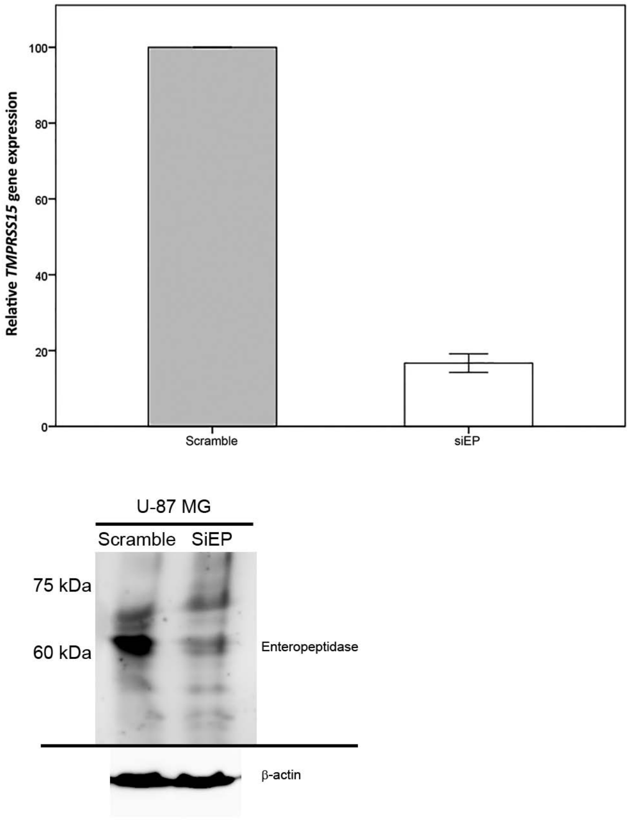
Relative *TMPRSS15* gene expression
100
80
60
40
20
0
Scramble
siEP
U-87 MG
Scramble
SiEP
75 kDa
60 kDa
Enteropeptidase
β-actin
